# OdonTraits Europe. A comprehensive traits dataset for European dragonflies and damselflies

**DOI:** 10.1038/s41597-026-07667-5

**Published:** 2026-06-27

**Authors:** Greet De Knijf, Jason Bried, Thore Engel, Martin Jeanmougin, Colin Fontaine, Reto Schmucki, Lisa Nicvert

**Affiliations:** 1 Research Institute for Nature and Forest (INBO), Havenlaan 88 bus 73, 1000 Brussels, Belgium; 2 Illinois Natural History Survey, Prairie Research Institute, University of Illinois Urbana-Champaign, Champaign, Illinois USA; 3 Institute of Biodiversity, Friedrich Schiller University Jena, Jena, Germany; 4 Department of Biodiversity and People, Helmholtz Centre for Environmental Research - UFZ, Leipzig, Germany; 5 German Centre for Integrative Biodiversity Research (iDiv) Halle-Jena-Leipzig, Leipzig, Germany; 6 Centre d’Ecologie et des Sciences de la Conservation (CESCO), Muséum national d’Histoire naturelle, Centre National de la Recherche Scientifique, Sorbonne Université, CP 135, 57 rue Cuvier, 75005 Paris, France; 7 UK Centre for Ecology & Hydrology, Maclean Building, Benson Lane, Wallingford, OX10 8BB UK; 8 FRB-Cesab, Univ Montpellier, Montpellier, France

## Abstract

Species traits are an important facet of biodiversity and are useful for testing many ecological and evolutionary hypotheses. Many initiatives to centralize species traits have emerged in recent years, but there are still large gaps in species traits’ knowledge in the literature. Odonata (dragonflies and damselflies) are present in most freshwater and surrounding ecosystems and are important indicators of freshwater health and conditions across the land-water interface. They are also important predators and prey both as larvae and adults and are vital to land-water energy transfers and community functioning. Here we present OdonTraits Europe, a database aggregating traits of all 143 European resident Odonata species. Our database compiles 43 traits representing adult and larvae morphology, life history, behaviour, phenology, and other ecological attributes, along with species legal, endemism, and conservation status, with a taxonomic coverage of >95% for all traits. Accessible and robust coverage of Odonata species traits will help to advance knowledge and applications involving this sentinel of the freshwater realm.

## Background & Summary

Information on biodiversity is becoming more widespread thanks to open science initiatives, data digitization, and data sharing between researchers and institutes^1^. However, comprehensive tools gathering this information are lacking or face important knowledge gaps.

Trait data are of importance for ecological, biogeographical, evolutionary and conservation research projects. Traits can be defined as “any morphological, physiological or phenological feature measurable at the individual level, from cell to whole-organism level, without reference to the environment or any other level of organization”^2^. Traits can also be based on ecological preferences like habitat and estimated from species distributions^3^. All those components of biodiversity reflecting species identity in a community and how an ecosystem operates or functions can be referred to as functional traits^4,5^.

Dragonflies (suborder Anisoptera) and damselflies (suborder Zygoptera), commonly known as dragonflies or odonates, are a key component of freshwater and adjacent ecosystems. They serve as model organisms in many areas of basic and applied ecological research and are important predators and prey both as larvae and adults^6^. As many species are sensitive to anthropogenic environmental pressures and the quality of streams, rivers and standing waters, a large proportion are threatened^7^. Dragonflies have been especially valuable for assessing aquatic and land-water conditions and are widely considered as good bioindicators and sentinels of the freshwater realm^8^.

Efforts to compile and publish Odonata traits are increasing, albeit with limited geographic and taxonomic scope. For instance, the Odonata Phenotypic Database^9^, which aims at a complete coverage for all Odonata globally yet is still largely incomplete (16% taxonomic coverage). For species occurring in the Brazilian Amazon, there is the Amazonian Odonata trait bank^10^. The first European traits database for dragonflies^11^ contained information for the 43 species present in the UK. For 79 odonate species known in Central Europe, an ambitious traits database was developed^12^, including rarely available traits such as body weight, flight height, egg clutch size, and egg width. Despite the scarce data and limited taxonomic coverage for such traits, this is the most widely used traits database for dragonflies in Europe. More recently, a trait database for the 84 dragonfly species known in Iberia and Morocco was published^13^. Although such databases are reliable in their respective regions, the trait values may differ in other regions due to local adaptation and phenotypic plasticity. Reliable trait coverage across Europe requires either a coordinated network of standardized regional databases or a centralized large database at that scale. The latter is more readily achievable, benefits a wider range of end users, and bypasses aggregation steps for multi-regional and continental applications.

Although dragonflies have been the subject of numerous ecological and conservation studies in Europe, there is no comprehensive traits database for all European species. Here, we present the OdonTraits Europe database, which compiles traits for 143 European species assessed in the European Red List (ERL)^14^, which was the starting point for the creation of this database. For each of the species assessed in the ERL, we compiled ecological, biological and morphological traits both for larvae and adults, together with their conservation and protection status from the best available knowledge at the European scale.

The aim of this work is to provide the most up-to-date comprehensive tool and database to anyone wishing to use traits information on Odonata at the European level. Diversity of traits information for all resident dragonflies and damselflies present in Europe are compiled and presented in an effective machine-readable form ready for analyses. The OdonTraits Europe database will serve as a reference for a wide range of evolutionary, ecological, and conservation studies dealing with dragonflies and damselflies in Europe, especially at the international and continental scales.

## Methods

We compiled a database covering 43 traits for all dragonflies and damselflies having resident populations in Europe, using the 2024 European Red List^14^ as the taxonomic backbone. These traits cover a broad spectrum of biological, morphological and ecological attributes, together with threat and conservation status.

This database is a synthesis of the available literature published in books, reports, atlas projects and scientific articles. Main sources of information on biological and ecological traits were fieldguides^15–18^, atlasses^19,20^, books on ecology of European species^21–24^, and papers on recently discovered species in Europe^25–27^. Unpublished ecological information on the recently described *Onychogomphus cazuma* was provided by C. Díaz-Martínez. Information on Species Temperature Index is mainly based on published work^28^, but also includes non-published information by the authors or from European experts. We further included biological and morphological traits of exuvia^29,30^, supplemented with unpublished material from C. Brochard. Our database also includes information on the legal protection status, endemism^31^ and the Red List status in Europe^14^. We aim to start an open continent-wide database that will expand over time with newly published sources and unpublished information from European experts.

### Ethics statement

This manuscript did not involve any experimental work.

## Data Record

OdonTraits Europe is available as open access on Zenodo^32^. Data were collated for European species following the last IUCN Red List assessment^14^ for a total 143 species that have known resident populations in Europe; excludes species known only from accidental, vagrant, or migratory records.

Our database covers the European continent, extending from Iceland, Svalbard and Franz Josef Land in the north to the Canary Islands in the south, and from the Azores in the west to the Urals in the east, including the European part of Türkiye and most of the European parts of the Russian Federation, Cyprus, and the European Macaronesian islands (the Canaries, Madeiran and Azores archipelagos).

**Fig. 1:**
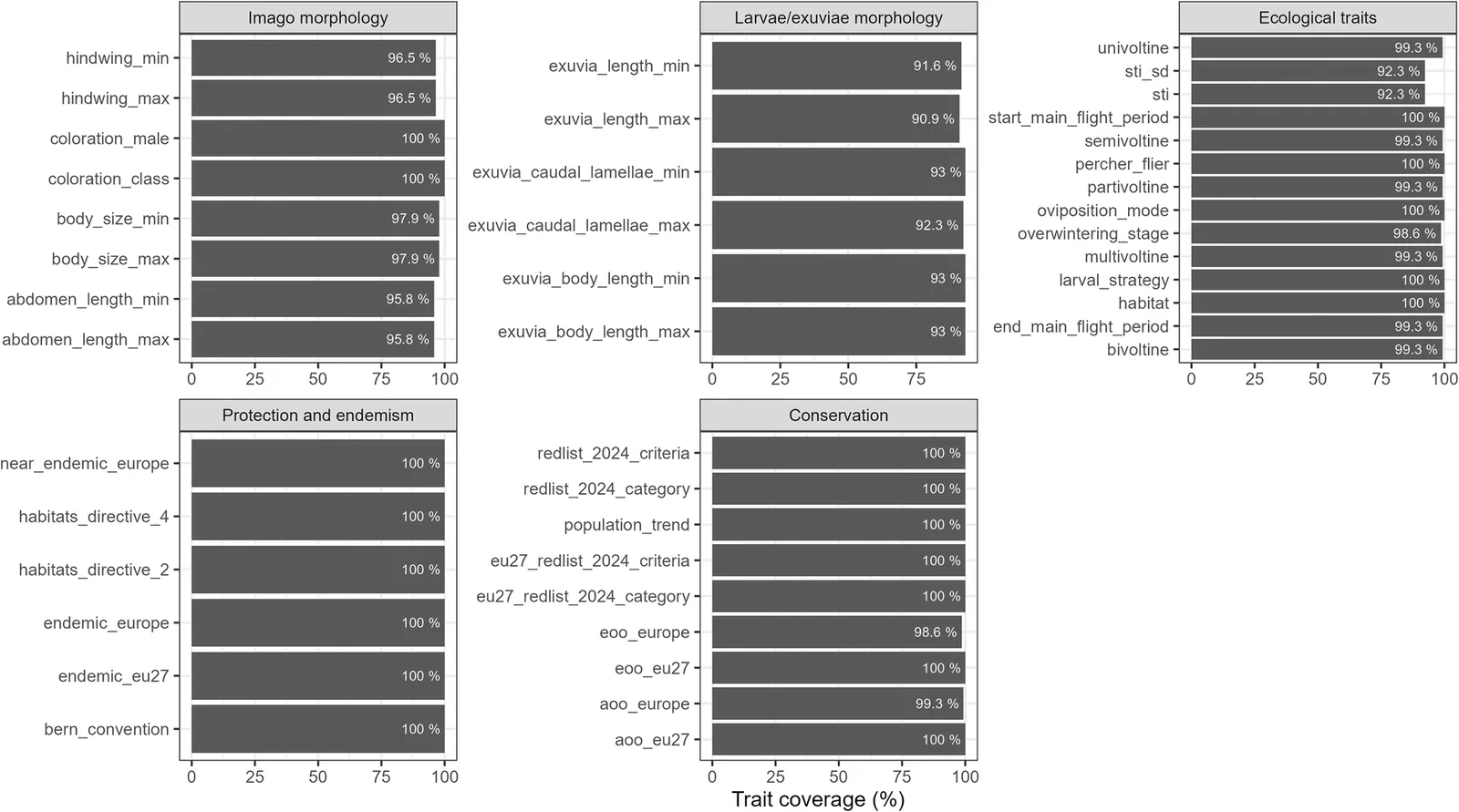
Traits coverage from the OdonTraits Europe database. Each panel corresponds to a section of the database, corresponding to a category of traits. The y-axis displays traits as they are named in the database, and the x-axis gives the coverage for each trait (in percentage of species).

The database OdonTraits Europe consists of eight files in CSV format. There are five main sections detailed below covering (1) adult and (2) larval morphology, (3) ecological attributes, (4) legal protection and endemism, and (5) conservation status indicators (see Fig. 1). Three additional tables gather taxonomic information (scientific name, naming authority, family), trait data sources, and associated references.

Morphological traits (Table 1) of adults (imago) include total body size (i.e. head, thorax and abdomen; min & max to account for sexual dimorphism), abdomen length (min & max), hind wing length (min & max), male coloration and coloration class (dark, intermediate, pale).

**Table 1:** Morphological traits of Odonata adults in Europe.

Column label	Column Description
body_size_min	minimal adult body size in millimeters
body_size_max	maximal adult body size in millimeters
abdomen_length_min	minimal abdomen length in millimeters
abdomen_length_max	maximal abdomen length in millimeters
hindwing_min	minimal hindwing span in millimeters
hindwing_max	maximal hindwing span in millimeters
coloration_male	specific coloration of mature male body: 1 - dark/black; 2 - azure blue/black; 3 - metallic green/blue; 4 - yellow/dark; 5 - brownish; 6 - green-yellow; 7 - gray-blue; 8 - red
coloration_class	general aspect of mature male body coloration: dark (black, azure-blue, brown, dark green); intermediate; pale (red, yellow, light blue)

Morphological traits of final-instar (F-0) larvae (Table 2) include exuvia total length (min & max, body + caudal lamellae), which is further split up for Zygoptera into body length (min & max, excluding caudal lamellae) and caudal lamellae length (min & max).

**Table 2:** Morphological traits of final-instar Odonata larvae (measured from final exuvia) in Europe.

Column label	Column Description
exuvia_length_min	minimal exuvia total length in millimeters (including caudal lamellae for Zygoptera)
exuvia_length_max	maximal exuvia total length in millimeters (including caudal lamellae for Zygoptera)
exuvia_body_length_min	minimal exuvia body length (mm) for Zygoptera only
exuvia_body_length_max	maximal exuvia body length (mm) for Zygoptera only
exuvia_caudal_lamellae_min	minimal exuvia length of caudal lamellae (mm) for Zygoptera only
exuvia_caudal_lamellae_max	maximal exuvia length of caudal lamellae (mm) for Zygoptera only

Life history, behavioral, phenological, and other ecological traits (Table 3) include species’ habitat preference, percher versus flier activity mode, start of main flight period, end of main flight period, Species Temperature Index (STI) and STI standard deviation, primary voltinism (partivoltine, semivoltine, univoltine, bivoltine, multivoltine), overwintering stage (egg, larva or adult), oviposition substrate (endophytic, exophytic, sediment), and larval strategy (burrower, clasper, hider, sprawler).

**Table 3:** Ecological traits of Odonata in Europe.

Column label	Column Description
habitat	habitat preference: 1 - eutrophic; 2 - oligotrophic; 3 - temporary waters; 4 - streams and rivers; 5 - southern streams and rivers ( = Mediterranean); 6 - generalist (species occurring in a wide range of standing and flowing waters)
percher_flier	adult activity modes related to thermoregulation and foraging, where perchers spend most time at rest, fliers spend more time in flight, and a flier_percher uses both strategies
start_main_flight_period	start of the main flight period in Europe, excluding the extreme early records
end_main_flight_period	end of the main flight period in Europe, excluding the extreme late records
sti	Species Temperature Index (STI) is the average temperature (°C) of the species’ range in Europe, based on the data from 1990 onwards from the European atlas; uses average monthly temperatures for 1960–1990 (WorldClim) at a 50 × 50 km grid scale.
sti_sd	Species Temperature Index (STI) standard deviation
partivoltine	>2 years to complete one generation
semivoltine	2 years to complete one generation
univoltine	1 year to complete one generation
bivoltine	2 generations completed in one year
multivoltine	>2 generations completed in one year
overwintering_stage	overwintering as: larva; egg; egg_larva (semi- or partivoltine species overwinters first year in egg stadium and second winter as larva); adult
oviposition_mode	endophytic = the laying of eggs inside a substrate, usually living or dead plant tissue; exophytic = the dropping of eggs onto the water surface or during flight; sediment = the laying of eggs inside the bottom substrate of a stream
larval_strategy	position in the water of the larvae (behaviour and feeding strategy): burrower = in the bottom sediment; clasper = on rocks, stones, soil; hider = hide within roots of trees; sprawler = within the submerged vegetation

Legal protection and endemism status (Table 4) acknowledges if a species is listed in the Bern Convention and mentioned on the Annexes of the European Habitats Directive, and if a species is endemic or near-endemic to Europe or the European Union countries (EU27).

**Table 4:** Legal protection and endemism of Odonata in Europe.

Column label	Column Description
bern_convention	Species listed in the Council of Europe’s Convention on the Conservation of European Wildlife and Natural Habitats (1979), known as the Bern Convention, which was the first legal convention to protect Europe’s wild plants and animals.
habitats_directive_2	Species mentioned on Annex II of the European Habitats Directive; countries need to designate, protect and manage special areas of conservation for those species
habitats_directive_4	Species mentioned on Annex IV of the European Habitats Directive; species of “community interest” that requires strict protection throughout its entire range
endemic_europe	Species is endemic to Europe
endemic_eu27	Species is endemic to the 27 countries of the European Union (EU27)
near_endemic_europe	Species just occurs marginally outside Europe, i.e. North Africa, Turkey or just east of the Ural Mountains

Conservation status indicators (Table 5) include the species IUCN Red List category and qualifying criteria for threatened or near-threatened status, for Europe and EU27, and the species’ population trend, extent of occurrence (EOO) and area of occupancy (AOO), the latter two both for Europe and EU27.

**Table 5:** Conservation status indicators of Odonata in Europe.

Column label	Column Description
redlist_2024_category	Red list category in Europe as assessed in the European Red List in 2024: Critically Endangered (CR); Endangered (EN); Vulnerable (VU); Near Threatened (NT); Data Deficient (DD); Least Concern (LC); Not Applicable (NA) (species does not occur in the focal region)
redlist_2024_criteria	IUCN Red List criteria for threatened (CR, EN, VU) or near-threatened (NT) status
eu27_redlist_2024_category	Red list category in EU27 as assessed in the European Red List in 2024: Critically Endangered (CR); Endangered (EN); Vulnerable (VU); Near Threatened (NT); Data Deficient (DD); Least Concern (LC); Not Applicable (NA) (species does not occur in the focal region)
eu27_redlist_2024_criteria	IUCN Red List criteria for threatened (CR, EN, VU) or near-threatened (NT) status
population_trend	Occurrence trends of dragonflies in Europe over the period 2010–2020, as calculated for the European Red List: decreasing; increasing; stable; unknown
eoo_europe	Extent of Occurrence (EOO) of a species in Europe in km² in the period 2000–2020, calculated using IUCN method; this is the surface area of a convex hull around the records and includes not suitable habitats for the species and even seas
aoo_europe	Area of Occupancy (AOO) is the area within its extent of occurrence in Europe which is occupied by a species, calculated here as the sum of all 2 × 2 km squares where the species has been found in the period 2000–2020
eoo_eu27	Extent of Occurrence (EOO) of a species in EU27 in km² in the period 2000–2020, calculated using IUCN method; this is the surface area of a convex hull around the records and includes not suitable habitats for the species and even seas
aoo_eu27	Area of Occupancy (AOO) is the area within its ‘extent of occurrence’ in EU27 which is occupied by a species, calculated here as the sum of all 2 × 2 km squares where the species has been found in the period 2000–2020

## Data Overview

**Fig. 2:**
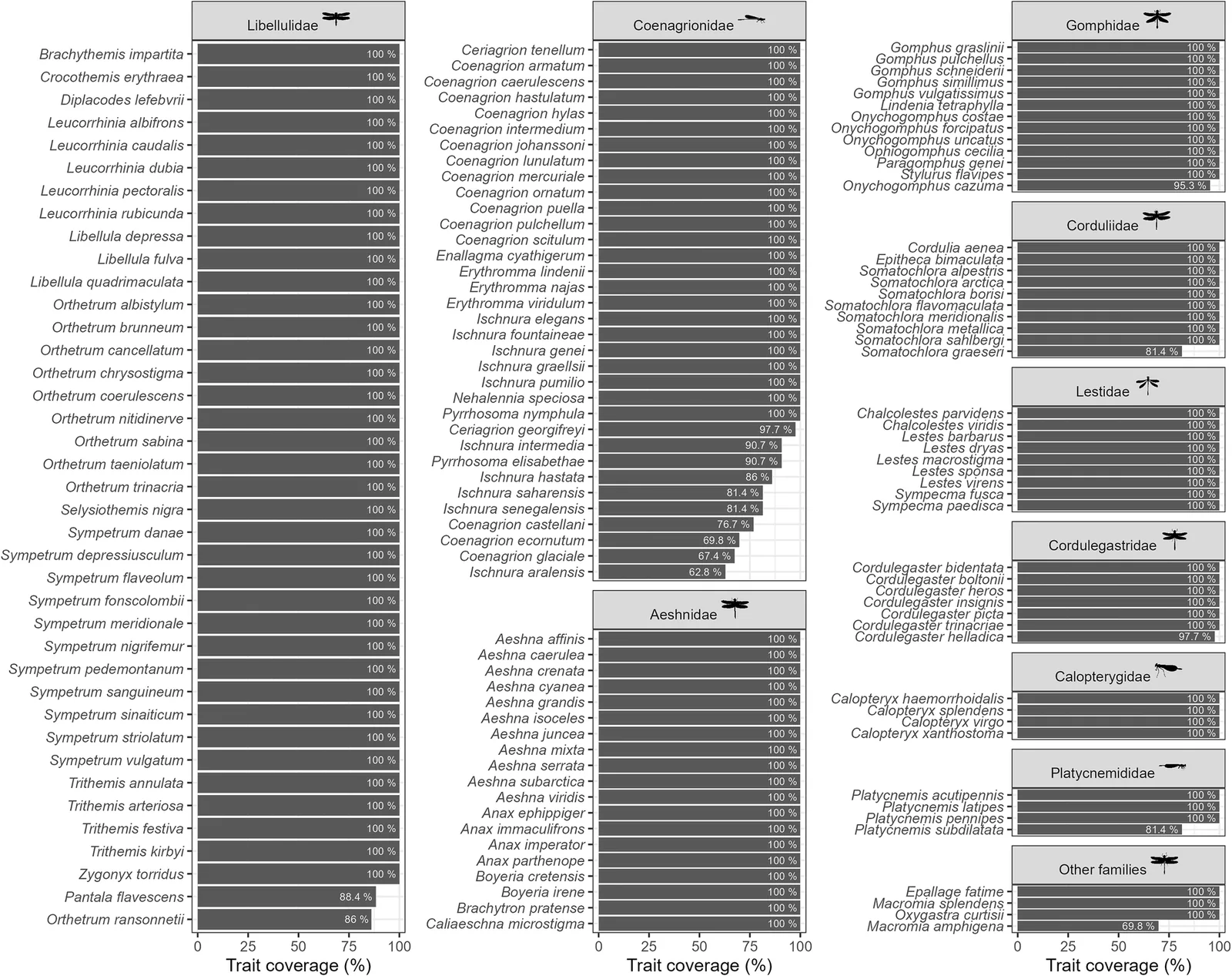
Trait coverage for each species in the OdonTraits Europe database. Each panel corresponds to a family; *Oxygastra curtisii* was traditionally placed under Corduliidae but its taxonomy is presently uncertain. The y-axis shows species names, and the x-axis gives the trait coverage for each species (in percentage of traits). Organism silhouettes are from PhyloPic and were contributed by Andy Wilson, Lisa Nicvert, Wouter Koch, Gareth Monger, Pascal Abel, and Maxime Dahirel.

OdonTraits Europe has a high trait coverage: all traits have complete information for more than 90% of species (Fig. 1), and the vast majority of species have a 100% traits coverage (Fig. 2).

## Technical Validation

All information contained in the database was double-checked by dragonfly experts. To ensure data formatting consistency, we also formatted and checked traits values using an automated pipeline written in R (https://github.com/Dragon-odonates/odontraitseurope).

## Data Availability

The complete dataset is available on Zenodo^32^ at the following link: https://doi.org/10.5281/zenodo.17248815.

## Code availability

Code to reproduce the figures (with R 4.5.1 and ggplot2) is available on GitHub https://github.com/Dragon-odonates/odontraitseurope.

## References

[1] Ball-Damerow, J. E. *et al*. Research applications of primary biodiversity databases in the digital age. *PLoS ONE* **14**(9), e0215794, 10.1371/journal.pone.0215794 (2019). PMC6738577 31509534

[2] Violle, C. *et al*. Let the concept of trait be functional! *Oikos* **16**(5), 882–892, 10.1111/j.0030-1299.2007.15559.x (2007).

[3] Chichorro, F., Juslén, A. & Cardoso, P. A review of the relation between species traits and extinction risk. *Biological Conservation* **237**, 220–229, 10.1016/j.biocon.2019.07.001 (2019).

[4] Petchey, O. L. & Gaston, K. J. Functional Diversity: Back to Basics and Looking Forward. *Ecology Letters* **9**(6), 741–758, 10.1111/j.1461-0248.2006.00924.x (2006). 16706917

[5] Schmera, D., Heino, J., Podani, J., Erős, T. & Dolédec, S. Functional Diversity: A Review of Methodology and Current Knowledge in Freshwater Macroinvertebrate Research. *Hydrobiologia* **787**, 27–44, 10.1007/s10750-016-2974-5 (2017).

[6] Córdoba-Aguilar, A., Beatty, C. D. & Bried, J. T. *Dragonflies & Damselflies: Model Organisms for Ecological and Evolutionary Research 2nd edn* (Oxford University Press, 2023).

[7] Sayer, C. A. *et al*. One-quarter of freshwater fauna threatened with extinction. *Nature* **638**, 138–145, 10.1038/s41586-024-08375-z (2025). PMC11798842 39779863

[8] Samways, M. J. *Conservation of dragonflies: sentinels for freshwater conservation* 10.1079/9781789248395.0007 (CABI/Royal Entomological Society, 2024).

[9] Waller, J. T., Willink, B., Tschol, M. & Svensson, E. I. The odonate phenotypic database, a new open data resource for comparative studies of an old insect order. *Scientific Data* **6**, 316, 10.1038/s41597-019-0318-9 (2019). PMC6908694 31831730

[10] Ferreira, V. R. S. *et al*. Amazonian Odonata Trait Bank. *Ecology and Evolution* **13**(6), e10149, 10.1002/ece3.10149 (2023). PMC10271597 37332521

[11] Powney, G. D. *et al*. Morphological and geographical traits of the British Odonata. *Data paper. Biodiversity Data Journal* **2**, e1041, 10.3897/BDJ.2.e1041 (2014). PMC4030211 24855438

[12] Harabiš, F. & Hronková, J. European database of the life-history, morphological and habitat characteristics of dragonflies (Odonata). *European Journal of Entomology* **117**, 302–308, 10.14411/eje.2020.035 (2020).

[13] Viza, A. *et al*. Species-specific functional traits rather than phylogenetic relatedness better predict future range-shift responses of odonates. *Insect Conservation and Diversity* **16**(5), 574–587, 10.1111/icad.12647 (2023).

[14] De Knijf, G. *et al*. *Measuring the pulse of European biodiversity. European Red List of Dragonflies & Damselflies (Odonata)* 10.2779/462053 (European Commission Brussels, 2024).

[15] Billqvist, M., Andersson, D. & Bergendorff, C. *Nordens trollsländor. Ny och utökad upplaga* (Avium Förlag AB, 2023).

[16] Dijkstra, K.-D. B., Schröter, A. & Lewington, R. *Field guide to the dragonflies of Britain and Europe 2nd edn* (Bloomsbury Publishing, 2020).

[17] Dijkstra, K.-D. B., Assandri, G. & Galimberti, A. Morphological and molecular evidence supports the species status of the Italian endemic *Coenagrion castellani* Roberts, 1948 (Coenagrionidae). *International Journal of Odonatology* **26**, 44–53, 10.48156/1388.2023.1917025 (2023).

[18] Smallshire, D. S. & Wash, A. Europe’s Dragonflies. *A field Guide to the damselflies and dragonflies* (Princeton University Press & British Dragonfly Society, 2020).

[19] Boudot, J.-P. & Kalkman, V. J. *Atlas of the Dragonflies and Damselflies of Europe* (KNNV Publishing, 2015).

[20] Boudot, J.-P. *et al*. Atlas of the dragonflies and damselflies of West and Central Asia. *Brachytron* **22**(Supplement), 4–238 (2021).

[21] Sternberg, K. & Buchwald, R. *Die Libellen Baden-Württembergs. Band 1: Allgemeiner Teil, Kleinlibellen (Zygoptera)* (Ulmer Verlag, 1999).

[22] Sternberg, K. & Buchwald, R. *Die Libellen Baden-Württembergs. Band 2: Grosslibellen (Anisoptera)* (Ulmer Verlag, 2000).

[23] Suhling, F. & Müller, O. *Die Flußjungfern Europas: Gomphidae* (Neue Brehm-Bücherei, 1996).

[24] Wildermuth, H. & Martens, A. *Die Libellen Europas. Alle Arten von der Azoren bis zum Ural im Porträt* (Quelle & Meyer Verlag, 2019).

[25] De Knijf, G. *et al*. Distribution, ecology and status of a threatened species *Ischnura intermedia* (Insecta: Odonata), new for Europe. *International Journal of Odonatology* **19**, 257–274, 10.1080/13887890.2016.1259662 (2016).

[26] Lopez-Estrada, E. K., Fernandez, J. B., Cardo-Maeso, N., Montejano, S. T. & Díaz-Martínez, C. *Onychogomphus cazuma* sp. nov. from Spain: Molecular and morphological evidence supports the discovery of a new European dragonfly species (Odonata: Gomphidae). *Odonatologica* **49**, 125–154, 10.5281/zenodo.3823337 (2020).

[27] Sparrow, D. J., De Knijf, G. & Sparrow, R. Diversity, status and phenology of the dragonflies and damselflies of Cyprus (Insecta: Odonata). *Diversity* **13**(11), 532, 10.3390/d13110532 (2021).

[28] Termaat, T. *et al*. Vries, M. F. Distribution trends of European dragonflies under climate change. *Diversity and Distributions* **25**(6), 936–950, 10.1111/ddi.12913 (2019).

[29] Brochard, C. *et al*. *Libellen* (KNNV Uitgeverij, 2012).

[30] Brochard, C. & van der Ploeg, E. *Fotogids Larven van Libellen* (KNNV Uitgeverij, 2014).

[31] De Knijf, G., Termaat, T. & Ott, J. Conservation of European dragonflies and damselflies in Atlas of the Dragonflies and Damselflies of Europe. (ed. Boudot, J.-P. & Kalkman, V. J.) (KNNV Publishing, 2015).

[32] De Knijf, G. *et al*. OdonTraits Europe. A comprehensive traits dataset for European dragonflies and damselflies. *Zenodo*10.5281/zenodo.17248815 (2026). PMC13694265 42373662

